# ε-Aminocaproic acid does not increase adverse effects in cardiac surgery: an analysis of 2,852 cases

**DOI:** 10.1186/cc13294

**Published:** 2014-03-17

**Authors:** S Zefferino, L Camara, J Almeida, F Galas, L Camara, J Auler, J Fukushima, A Lema, L Hajjar

**Affiliations:** 1 Heart Institute, São Paulo, Brazil; 2 Instituto do Cancer do Estado de São Paulo, Brazil

## Introduction

Antifibrinolytic drugs recently have been associated with adverse outcomes in patients undergoing cardiac surgery. We reviewed our experience with prophylatic ε-aminocaproic acid in patients undergoing cardiac surgery at InCor - Heart Institute.

## Methods

We retrieved data on 2,852 consecutive patients undergoing cardiac surgery at revascularization at Duke between 1 January 2004 and 31 December 2008. We compared patients receiving or not prophylatic ε-aminocaproic acid in coronary artery bypass graft surgery, or valve procedures or combined ones. We evaluated baseline characteristics, intraoperative data and severe clinical endpoints during 30 days.

## Results

A total of 2,852 patients were included in the analysis and 1,389 (48.7%) received prophylactic ε-aminocaproic acid. The others did not receive any antifibrinolytic. In the risk-adjusted model, survival was similar among patients treated with aminocaproic acid or not treated (1.9 vs. 1.6%, *P *= 0.48). Bleeding in 24 hours was reduced in the treated group as compared with the nontreated (391 vs. 472 ml, *P *= 0.012). There were no differences regarding acute renal failure, stroke and infection in 30 days.

## Conclusion

In cardiac surgery, prophylactic use of aminocaproic acid reduces bleeding and does not result in higher incidence of acute complications.

